# Clinical Research Informatics: Contributions from 2024

**DOI:** 10.1055/s-0046-1816614

**Published:** 2026-11-03

**Authors:** Xavier Tannier, Dipak Kalra, Pascal Coorevits

**Affiliations:** 1 Sorbonne University, Inserm, University Sorbonne Paris-Nord, Limics, F-75006 ParisFrance; 2 Ghent University, Faculty of Medicine and Health Sciences, Department of Public Health and Primary Care, Unit of Medical Informatics and Statistics, GhentBelgium

**Keywords:** International Medical Informatics Association Yearbook, Clinical trials, Electronic health record, Bias

## Abstract

**Objectives** : To summarize key contributions to research in Clinical Research Informatics (CRI) and to select the best papers published in 2024.

**Method** : A bibliographic search using a combination of MeSH descriptors and free-text terms on CRI was performed using PubMed, followed by a double-blind review to select a list of candidate best papers to be then peer-reviewed by external reviewers. After peer-review ranking, a consensus meeting between the three section editors and the editorial team was organized to finally conclude on the selected two best papers.

**Results** : Among the 1417 papers returned by the search, published in 2024, that were in the scope of the various areas of CRI, the full review process selected two best papers. The first best paper investigates how to identify and mitigate group biases—such as subgroup underrepresentation—in computational phenotyping. The second-best paper addresses group bias in clinical trials by examining how restrictive eligibility criteria and enrollment challenges affect patient representativeness at both the population and subgroup levels. This year's survey paper is a scoping review related to digital health and environmental sustainability, highlighting the urgent need to embed sustainable practices across the technology lifecycle and the critical regulatory gaps in addressing environmental impacts.

**Conclusions** : The literature relevant to CRI in 2024 highlights key themes including bias and representativeness, quality and interoperability, and advances in phenotyping as well as real-world evidence extraction and generation.

## 1. Introduction

Within the 2024 International Medical Informatics Association (IMIA) Yearbook, the goal of the Clinical Research Informatics (CRI) section is to provide an overview of research trends from 2024 publications that demonstrate the progress in multifaceted aspects of medical informatics supporting research and innovation in the healthcare domain. New methods, tools, and CRI systems have been developed to enable real-world evidence generation and optimize the life-cycle of clinical trials. Although thisxs year's Yearbook focus should ideally highlight environmental issues, we found that too few papers explicitly addressed this perspective within the context of clinical research informatics.

## 2. About the paper Selection

A comprehensive review of articles published in 2024 and addressing a wide range of issues for CRI was conducted. The selection was performed by querying MEDLINE via PubMed (from NCBI, National Center for Biotechnology Information) with a set of predefined MeSH descriptors and free terms: Clinical research informatics, Biomedical research, Nursing research, Clinical research, Medical research, Pharmacovigilance, Patient selection, Phenotyping, Genotype-phenotype associations, Feasibility studies, Eligibility criteria, Feasibility criteria, Cohort selection, Patient recruitment, Clinical trial eligibility screening, Eligibility determination, Patient-trial matching, Protocol feasibility, Real world evidence, Data Collection, Epidemiologic research design, Clinical studies as Topic, Multicenter studies as Topic, and Evaluation studies as Topic. Review papers, as well as papers addressing topics of other sections of the Yearbook, such as Translational Bioinformatics, were excluded based on the predefined exclusion of MeSH descriptors such as Genetic research, Gene ontology, Human genome project, Stem cell research, or Molecular epidemiology.

Bibliographic databases were searched twice, initially in November 2024 and then refreshed for late in the year publications on 9th January 2025, considering the electronic publication date. 1417 papers were found and screened for relevance to CRI, for reporting original research as opposed to opinion or educational papers, and their scientific quality was blindly rated as low, medium, high or out of scope by the three section editors based on papers' title and abstract. 41 references classified as high to medium quality contributions to the field by at least two section editors were considered and classified into the following areas of the CRI domain in order of the number of matching papers (multiple classification choices were permitted): reuse of EHR data, Real-World Evidence generation, electronic phenotyping; data integration, semantic interoperability and data quality assessment; security, confidentiality and data privacy; data/text mining, Artificial Intelligence and Machine Learning, communicating study results; feasibility studies, patient recruitment, data management, CRI systems and improved use experiences; ethical, legal, social, policy issues and solutions, stakeholder participation; research networks, team science, Learning Healthcare System, Health Technology Assessment. A further review by both editors after full paper screening resulted in 12 candidate papers being taken forward to external peer review. In conformance with the IMIA Yearbook process, these 12 papers were peer-reviewed by the IMIA Yearbook editors and external reviewers. Each paper was reviewed by 5 or 6 reviewers. Two papers were finally selected as best papers ( Table 1 ). A content summary of these best papers can be found in the appendix of this synopsis.

**Table 1.** Selection of best papers for the 2025 IMIA Yearbook of Medical Informatics for the section Clinical Research Informatics.

Section: Clinical Research Informatics
• Ding S, Zhang S, Hu X, Zou N. Identify and mitigate bias in electronic phenotyping: A comprehensive study from computational perspective. J. Biomed. Inform. 156:104671. 2024 Jun. https://doi.org/10.1016/j.jbi.2024.104671 . • Benedum CM, Sarkar S, Bozkurt S, Bhagat R, Richie N, Lavery B, Griffith SD. POP-REFINE: A Comprehensive Framework for Evaluating and Optimizing Representativeness in Clinical Trials. Clin. Pharm. Therap. 2024 Dec. https://doi.org/10.1002/cpt.3543 .

## 3. Outlook

The 12 candidate best papers for 2024 reflect the continued maturation of data-driven research in Clinical Research Informatics (CRI). This year's selections highlight key themes such as AI bias and data representativeness, semantic interoperability and data quality, as well as scalable phenotyping and real-world evidence generation. Together, these papers exemplify sustained efforts to ensure fairness, inclusivity, and reproducibility in the use of health data, while also exploring the potential of emerging technologies such as large language models and privacy-preserving knowledge graphs.

### 3.1 Bias and representativeness

The accelerated development and increasing adoption of AI, not only in health, has highlighted the errors and dangers arising from data bias that occur during initial training and validation of algorithms. This has heightened interest across countries and stakeholders in the importance of data quality and for diligent verification of the representativeness of health data sets before they are utilised for AI training and subsequently monitoring for bias during machine learning and when algorithms generate outputs. This has been a strong theme within the CRI literature over the past year, and four strong papers were identified for this synopsis, two of them having been nominated as the best CRI papers this year. Ding et al focus on electronic phenotyping [ 1 ]. This is a well-established methodology documented in previous yearbooks to derive the presence or absence of clinical characteristics of interest within electronic health records and based on multiple confirmatory or non-confirmatory data items in the same patient record. A bias in the phenotyping algorithm will lead to bias in the profile of the derived characteristics, leading downstream to incorrect (biased) inferences from the data. Their research examined several methods for bias mitigation at different points in the AI development life-cycle, of which it seems that patient data resampling is the most effective. More details about this best paper can be found in the Appendix.

AI bias was also examined by Griffin et al. [ 2 ], who have provided an overview of the areas within the AI development and use lifecycle at which inclusion ethical issues might arise . This includes bias within data sources, as discussed above, also fairness within algorithm development and in the handling of uncertainty, explainability and its impact on equity. They also discuss the wider societal implications that can influence the ways in which bias could arise and offer recommendations on how this could be avoided.

The best paper by Benedum et al. [ 3 ], focused on clinical trial recruitment. It is well recognised that some sub-populations are under-represented, as discussed in previous issues of this yearbook. The authors have developed a formalised methodology for assessing representativeness within a recruitment pool of patients, and of determining ways in which the eligibility criteria could be adapted to become more inclusive. More details about this paper can also be found in the Appendix.

Ban Al-Sahab et al., look more broadly at the generation of real-world evidence from electronic health record information, and the areas of bias that can arise [ 4 ]. They start with a healthcare focus, exploring the inequity in access to healthcare, the healthcare services and treatment choices that are offered to different sub-populations. They also examine data documentation practices, the balance between structured and unstructured data and data quality that might be different for different sub-populations. The culture of clinical practice and the influence of insurance and billing requirements are both implicated within this study. The authors then turn to the research use of data, which itself might select data that is more homogeneous and structured, thereby amplifying the preceding healthcare and clinical documentation biases.

Although bias in AI behaviour has caught public and decision-maker attention, only in recent years, bias in EHR data and bias in clinical research are long-standing problems. The spotlight provided by AI is helpful if we look beyond the AI use of data and utilise the mitigations now arising across the board of the clinical research uses of health data.

### 3.2 Quality and interoperability

Combining data from multiple heterogeneous electronic health record systems, whether creating merged data sets or conducting federated analysis, remains challenging and is usually undertaken first by mapping the heterogeneous data to a single standardised representation. Two strong papers on this long-standing challenge are presented in this year's CRI chapter. The Observational Medical Outcomes Partnership) OMOP Common Data Model is a frequently used standardised representation for federated observational research. The scientific integrity of the federated analyses critically depends upon the consistency of the mappings made to OMOP. Reich et al. focus attention on the semantic consistency in term mapping [ 5 ]. Data can be mapped to OMOP using a vast array of clinical terminologies, with over 8 million concepts including medicines, conditions, procedures, physiological measurements etc. The challenge is correctly determining the most appropriate term when multiple terminologies are in play across a complex information model. The authors have developed an ontological framework linking the different fields within the OMOP Common Data Model to relevant terminological concepts, to reduce the risk of mapping error. The ontology has been downloaded and used many thousands of times, and the authors have established a commenting feedback mechanism as an additional quality assurance measure.

Another use of data mapping is the import of the EHR data into electronic case report forms (eCRF) used for clinical trials. This is traditionally done by manual transcription although other research is demonstrating the electronic transfer, albeit requiring a preliminary manual mapping process. Wang et al. have applied Large Language Model technology to this mapping challenge, comparing ChatGLM and Large Language Model Meta AI (LLaMA) to extract and map relevant data from an EHR system as an AI response to questions designed to populate the fields in an eCRF [ 6 ]. In a single centre evaluation of 63 patients, ChatGLM achieved a similar level of transcription accuracy to manual transcription, in about half the time. However structured data fields, especially medications, scored high whereas health status information extracted from free text proved a challenge with only 20% accuracy. Despite the small sample and limited findings, this evaluation highlights an important avenue for further research.

Another important challenge with scaling up research using real-world data is the data quality. Förster et al. examined 30 years of historic data at a German hospital, assessing the quality of over 2 million record entries [ 7 ]. They found that about 25% of these entries were unusable because of data value errors and inconsistencies. Having studied clinical workflows and data documentation practices, they highlighted the complexity of the clinical user interfaces, the time pressures under which clinical documentation takes place and a lack of training in data entry for the hospital information system were all contributors to the errors. A further factor was that staff sometimes had to adopt workarounds to enter data in unintended fields because there was no appropriate field for the information they needed to document. The authors implemented data cleaning processes to significantly improve the usability of the data but emphasize the importance of addressing the root causes.

Another study about the causes of poor data quality is reported by Fraser et al. [ 8 ], across 50 healthcare facilities in Rwanda using the OpenMRS EHR system. 28 HIV variables were extracted from paper records and OpenMRS, for a total of around 3500 patients. The centres had previously been stratified with half of them receiving a decision support alert to enter viral load data, the other half having no alerts. The findings demonstrated a reasonably good data quality across most data elements except for viral load and some treatment information. The centres having a reminder alert to complete the missing information had a significantly higher level of completeness for these data items. User training and feedback on data quality were also considered to be important quality improvement strategies.

### 3.3 Phenotyping and real-world evidence extraction and generation

Phenotyping and real-world evidence (RWE) extraction and generation are foundational for advancing personalized medicine and improving healthcare outcomes. Phenotyping involves identifying patient characteristics or conditions from clinical data, while RWE leverages data from routine clinical practice to inform decision-making. Both aspects enable scalable, data-driven insights and support the development of AI-powered tools, knowledge graphs, and interoperable systems that enhance clinical decision support and research.

Shang et al. have introduced a privacy-preserving, multicenter knowledge graph system that enables collaborative reasoning across fragmented Electronic Health Records [ 9 ]. Using a unified semantic structure and OMOP vocabulary, local data are transformed into intermediate reasoning outputs, which are anonymized and shared via blockchain. In a real-world application involving 1,185 patients across three hospitals, the system identified 124 chronic kidney disease cases that were undetectable using single-hospital data. Clinician validation confirmed 86% of these cases, demonstrating the system's potential to enhance diagnostic accuracy through federated data integration.

In a study of Erturkmen and colleagues, the European Patient Summary (EPS) and HL7 FHIR International Patient Summary Implementation Guide for their suitability in secondary use for cardiology research was assessed [ 10 ]. Analyzing two EU-funded projects - DataTools4Heart and AI4HF - the authors conducted a gap analysis comparing required data elements for AI model development with those supported by EPS. Findings suggest that with minor extensions, EPS can effectively support AI-driven research, advocating for a semantically interoperable core dataset to enhance cross-border research and precision medicine.

Beaton and colleagues evaluated patient portal messaging as a recruitment tool for the All of Us Research Program [ 11 ]. At Columbia University, over 59,000 patients were contacted, with 6.3% expressing interest and an estimated 2% enrolling. While underrepresented racial and ethnic groups were less likely to engage initially, they showed higher interest once they responded. This highlights the potential of digital outreach for inclusive recruitment, while emphasizing the need for tailored strategies to improve early-stage engagement.

In fourth study by Yan et al. [ 12 ], the use of large language models (LLMs) to automate the generation of SQL-based EHR phenotyping algorithms was investigated. GPT-4 and GPT-3.5 outperformed Claude 2 and Bard in logic and executability, though they struggled with balancing recall and precision. While LLMs can accelerate phenotyping by identifying relevant clinical concepts, expert oversight remains essential to refine and validate outputs for reliable use in observational research.
